# S2k guideline diagnosis and treatment of carbon monoxide poisoning

**DOI:** 10.3205/000300

**Published:** 2021-11-04

**Authors:** Björn Jüttner, Hans-Jörg Busch, Andreas Callies, Harald Dormann, Thorsten Janisch, Guido Kaiser, Hella Körner-Göbel, Karsten Kluba, Stefan Kluge, Bernd A. Leidel, Oliver Müller, Johannes Naser, Carsten Pohl, Karl Reiter, Dietmar Schneider, Enrico Staps, Wilhelm Welslau, Holger Wißuwa, Gabriele Wöbker, Cathleen Muche-Borowski

**Affiliations:** 1 German Interdisciplinary Association of Critical Care and Emergency Medicine (DIVI); 2 German Society of Medical Intensive Care and Emergency Medicine (DGIIN); 3 Bundesvereinigung der Arbeitsgemeinschaften der Notärzte Deutschlands (BAND); 4 German Association for Emergency Medicine (DGINA); 5 German Society of Anaesthesiology and Intensive Care Medicine (DGAI); 6 GIZ-Nord Poisons Center, University Medical Center Göttingen (GIZ-Nord); 7 Bundesverband der Ärztlichen Leiter Rettungsdienst Deutschland (ÄLRD); 8 The German Society of Anaesthesiology and Intensive Care Medicine (DGAI); 9 German Respiratory Society (DGP); 10 German Society for Diving and Hyperbaric Medicine (GTÜM); 11 AMEOS Klinikum Bernburg, Germany; 12 Society for Neonatology and Pediatric Intensive Care Medicine (GNPI); 13 German Society of NeuroIntensive Care and Emergency Medicine (DGNI); 14 Bundeswehrkrankenhaus Ulm, Germany; 15 The Association of the Scientific Medical Societies in Germany (AWMF)

**Keywords:** carbon monoxide poisoning, etiology, prevention, prehospital management, oxygen breathing, initial in-hospital care, hyperbaric oxygen therapy, HBOT, CO hemoglobin, delayed neurological sequelae (DNS), rehabilitation

## Abstract

Carbon monoxide (CO) can occur in numerous situations and ambient conditions, such as fire smoke, indoor fireplaces, silos containing large quantities of wood pellets, engine exhaust fumes, and when using hookahs.

Symptoms of CO poisoning are nonspecific and can range from dizziness, headache, and angina pectoris to unconsciousness and death.

This guideline presents the current state of knowledge and national recommendations on the diagnosis and treatment of patients with CO poisoning.

The diagnosis of CO poisoning is based on clinical symptoms and proven or probable exposure to CO. Negative carboxyhemoglobin (COHb) levels should not rule out CO poisoning if the history and symptoms are consistent with this phenomenon. Reduced oxygen-carrying capacity, impairment of the cellular respiratory chain, and immunomodulatory processes may result in myocardial and central nervous tissue damage even after a reduction in COHb.

If CO poisoning is suspected, 100% oxygen breathing should be immediately initiated in the prehospital setting.

Clinical symptoms do not correlate with COHb elimination from the blood; therefore, COHb monitoring alone is unsuitable for treatment management. Especially in the absence of improvement despite treatment, a reevaluation for other possible differential diagnoses ought to be performed.

Evidence regarding the benefit of hyperbaric oxygen therapy (HBOT) is scant and the subject of controversy due to the heterogeneity of studies.

If required, HBOT should be initiated within 6 h.

All patients with CO poisoning should be informed about the risk of delayed neurological sequelae (DNS).

## Zusammenfassung

Kohlenmonoxid (CO) kann in zahlreichen Situationen und Umgebungen auftreten, beispielsweise Brandrauch, Feuerstellen in geschlossenen Räumen, Silos mit großen Mengen an Holzpellets; Motoren-Abgase und der Gebrauch von Wasserpfeifen.

Die Symptome einer Kohlenmonoxidvergiftung sind unspezifisch und können Schwindel, Kopfschmerz, Angina pectoris bis zu Bewusstlosigkeit und Tod umfassen.

Diese Leitlinie legt den aktuellen Stand der Erkenntnisse und der nationalen Empfehlungen in der Diagnostik und Behandlung von Patienten mit Kohlenmonoxidvergiftungen dar.

Die Diagnose einer Kohlenmonoxidvergiftung erfordert klinische Symptome und eine nachgewiesene oder wahrscheinliche Exposition mit Kohlenmonoxid. Ein negativer CO-Hämoglobin (Hb)-Nachweis soll nicht zum Ausschluss einer Kohlenmonoxidvergiftung führen, wenn Anamnese und Symptome übereinstimmend sind. Durch eine reduzierte Sauerstofftransportkapazität, die Beeinträchtigung der zellulären Atmungskette und immunmodulatorische Prozesse kann es auch nach Reduktion des CO-Hb zu myokardialen und zentralnervösen Gewebeschäden kommen.

Bei Verdacht auf eine Kohlenmonoxidvergiftung soll präklinisch sofort mit einer 100% Sauerstoffatmung begonnen werden.

Die klinische Symptomatik der Patienten korreliert nicht mit der CO-Hb Clearance aus dem Blut. CO-Hb-Kontrollen allein sind für eine Therapiesteuerung ungeeignet. Insbesondere bei fehlender Besserung unter Therapie sollte eine Reevaluation für andere möglicherweise vorliegende Differentialdiagnosen erfolgen.

Die Evidenz zum Nutzen der hyperbaren Sauerstofftherapie (HBOT) ist aufgrund der heterogenen Studienlage niedrig und wird kontrovers diskutiert.

Der Beginn einer HBOT soll gegebenenfalls innerhalb von 6 Stunden erfolgen.

Jeder Patient mit Kohlenmonoxidvergiftung soll über das Risiko eines verzögert einsetzenden neurologischen Defizites (delayed neurological sequelae, DNS), aufgeklärt werden.

## 1 Introduction

### 1.1 Objective 

This guideline presents the current state of knowledge and recommendations on the diagnosis and treatment of patients with CO poisoning for the purposes of

- emergency first aid by medical assistants and physicians,
- the principles of clinical primary care,
- decision-making on primary or secondary transport to a hyperbaric oxygen therapy center,
- further medical care.

### 1.2 Methodological bases 

#### 1.2.1 Definitions used for strength of recommendations and consensus

##### 1.2.1.1 Formulation of the strength of recommendations

- Strong recommendation: should/should not
- Recommendation: ought to/ought not to
- Open recommendation: may be considered/no specific recommendation

##### 1.2.1.2 Classification of the strength of consensus

- Strong consensus: >95% of participants agree
- Consensus: >75–95% of participants agree
- Majority agreement: >50–75% of participants agree
- No consensus: <50% of participants agree

#### 1.2.2 Period of validity and updating procedure 

The S2k guideline will remain valid until the next update, with the validity period estimated to be 5 years. Regular updates are planned. Should urgent changes be required, these will be published separately. Comments and suggestions for the update process are expressly welcomed and can be sent to the following address: Deutsche Interdisziplinäre Vereinigung für Intensiv- und Notfallmedizin e.V., Luisenstr. 45, 10117 Berlin, Germany, info@divi-org.de.

## 2 Etiology and pathogenesis of carbon monoxide poisoning

### 2.1 Definition of carbon monoxide poisoning

**What is the definition of symptomatic CO poisoning?**

*CO poisoning is present when CO has been inhaled and corresponding symptoms occur (see Sect. 4).*

- Yes: 10, no: 0, abstention: 0
- Strength of consensus: 100% (strong consensus)

CO is an odorless and colorless gas with a similar molecular weight to that of air [1]. It is produced during incomplete combustion processes of substances containing carbon. Sources of exposure include: fires, faulty heating systems, fireplaces and stoves, inadequately ventilated garages, wood pellet stores, charcoal grills used indoors with suicidal intent, and hookah use [2], [3], [4].

Accidents in industrial facilities can result in significantly higher CO concentrations compared to residential environments.

Appropriate deployment codes and the use of portable CO detectors are intended to heighten the awareness of emergency responders [5] (see Sect. 3).

### 2.2 Pathophysiology 

**What are the main factors that influence the occurrence of symptomatic CO poisoning?**

*The concentration of CO in the breath, the duration of exposure, and the respiratory minute volume affect the amount ingested.*

*Pre-existing comorbidities may influence the symptoms of CO poisoning.*

- Yes: 10, no: 0, abstention: 0
- Strength of consensus: 100% (strong consensus)

CO rapidly diffuses across the alveolar membrane and binds preferentially to the heme iron moiety with an affinity approximately 230–300 times higher than that of oxygen [6]. Even low atmospheric concentrations of 10 ppm lead to measurable COHb values of around 2% [7].

A conformational change results in a left shift in the oxyhemoglobin dissociation curve, reduced oxygen transport capacity, and decreased delivery of oxygen to peripheral tissues. CO also binds in tissue to other heme-containing proteins, such as skeletal and myocardial myoglobin [8].

In addition, intracellular CO impairs the function of ferricytochrome enzymes such as cytochrome C oxidase [9]. The resulting tissue hypoxia manifests in the central nervous system, which is particularly sensitive to hypoxia, as well as in the heart muscle.

A single COHb reading correlates insufficiently with the severity of clinical manifestation [10], [11]. In addition to tissue hypoxia, CO leads to direct immunological and/or inflammatory damage at the cellular level. This results in, e.g., neutrophil activation, lymphocyte proliferation, mitochondrial dysfunction, and lipid peroxidation. Other major mechanisms of damage include oxygen radical formation, oxidative stress, inflammation, and apoptosis [8], [12], [13], [14].

### 2.3 Epidemiology

**What is the incidence of CO poisoning in Germany?**

*Only secondary data for inpatients are available from the Federal Statistical Office for a nationwide assessment. A reliable statement on incidence is not possible.*

- Yes: 8, no: 0, abstention: 0
- Strength of consensus: 100% (strong consensus)

Neither the United States (US) nor Germany keeps an active national registry of CO poisonings. The best available and comparable information based on secondary data show a total of 2,302 (0.8 per 100,000 inhabitants) non-fire-related registered cases of CO poisoning [15] for the US in 2007. In Germany, 3,943 inpatient cases (4.8 per 100,000 inhabitants) were registered in the same year (including fire-related poisoning) [16]. This information is provided nationwide by routine hospital data on full inpatients submitted to the health insurance funds according to § 301 SGB V (German Social code) or to the German Institute for the Hospital Remuneration System (InEK) according to § 21 of the German Hospital Remuneration Act (KHEntgG).

A significantly higher number of patients is presumed to be cared for in the outpatient setting. These cases are not recorded on a nationwide basis.

In the reported cause of death statistics from the German Federal Statistical Office, the number of deaths due to CO poisoning more than doubled between 2007 and 2018 (Table 1 (Tab. 1)). In 2018, 629 patients died in Germany as a result of CO poisoning (0.8 deaths per 100,000 inhabitants).

### 2.4 Prognosis

Mortality depends on CO exposure time as well as CO concentration and is significantly influenced by the toxicity of other gases involved [17].

In the further course following CO poisoning, there is a risk of delayed-onset neurocognitive impairment such as ataxia, memory loss, impaired concentration, behavioral problems, depression, anxiety, dyscalculia, and inner ear problems [8], [10], [18], [19], [20]. Structural changes to subcortical white matter and pallidum, as well as hippocampal atrophy, have been observed [21], [22], [23].

The severity of initial poisoning did not necessarily correlate with the development of long-term neuronal damage [24], [25].

Since long-term damage can occur after a symptom-free interval of days to weeks, the incidence of unreported cases is assumed to be high [10], [26], [27].

Patients with pre-existing coronary artery disease are at increased risk for myocardial infarction and arrhythmias [28].

Furthermore, a retrospective study of 230 patients following CO poisoning described an increase in cardiac biomarkers or electrocardiogram (ECG) changes in 37% of cases [29]. In the prospective study, myocardial damage was a significant predictor of mortality at the 7.6-year observation interval (adjusted hazard ratio [AHR]: 2.1; 95% confidence interval [1.2–3.7]; p=0.009). Age at poisoning also had an impact on mortality (AHR: 1.2 per 5-year increase in age [1.1–1.3]; p<0.001) [30].

Other retrospective cohort studies showed an association between CO poisoning and the occurrence of major cardiovascular events (AHR: 2.00 [1.83–2.18] or AHR: 1.83 [1.43–2.33]) [31], [32]. If comorbidities (diabetes mellitus, hypertension, and hyperlipoproteinemia) were present, the risk increased 14.7-fold [10.9–19.9] [32].

Prospective studies of children with CO poisoning describe varying frequencies of injury [33], [34]. Overall, children develop symptoms earlier compared to adults, but exhibit faster remission [35].

## 3 Prevention

**Which ambient conditions increase the likelihood of CO poisoning?**

*CO can occur in numerous situations and ambient conditions. Typical situations include fire smoke, loss of consciousness without apparent cause in enclosed spaces with fireplaces (e.g., heaters, stoves, fireplaces, barbecues); suicide (attempted) – often with cor* *re* *spond* *ing indicators (suicide note, taped-off rooms); in silos with large quantities of wood pellets; engine exhaust (without catalytic converter); and hookah use.*

- Yes: 8, no: 0, abstention: 0
- Strength of consensus: 100% (strong consensus)

**Which warning devices are suitable for the detection of CO exposure?**

*In the opinion of the guideline group, the use of early-warning detectors (smoke and CO alarms) in every household would be judicious.*

- Yes: 8, no: 0, abstention: 0
- Strength of consensus: 100% (strong consensus)

When the fire department responds to activated/sounding early-warning devices in residential buildings, it is not possible to distinguish between smoke and CO detectors on the basis of the acoustic signal before reaching the detector (e.g., activated detector in an apartment without evident signs of fire).

*The CO warning devices used by emergency responders help detect environments containing CO and should be deployed nationwide.*

- Yes: 8, no: 0, abstention: 0
- Strength of consensus: 100% (strong consensus)

## 4 Symptoms and diagnosis

**Is it possible to differentiate between acute, subacute, and chronic CO poisoning?**

*In the diagnosis and treatment of CO poisoning, it is usually not possible to distinguish between acute and chronic forms.*

- Yes: 8, no: 0, abstention: 0
- Strength of consensus: 100% (strong consensus)

CO poisoning can be acute or chronic (long-term exposure). It is unclear to what extent pathophysiological differences are significant here. The literature does not describe any distinctive symptomatology [8]. This guideline focuses on acute exposure. The effects of long-term exposure as in an occupational disease are not the subject of this guideline.

**Which investigative procedures are valid for the diagnosis and disease monitoring of CO poisoning?**

*The diagnosis of CO poisoning requires clinical symptoms and proven or probable exposure to CO.*

- Yes: 10, no: 0, abstention: 0
- Strength of consensus: 100% (strong consensus)

*The diagnosis ought to be made on the basis of clinical symptoms, patient history, the circumstances in which the patient is found, and symptoms.*

- Yes: 11, no: 0, abstention: 0
- Strength of consensus: 100% (strong consensus)

*Negative COHb levels should not result in the exclusion of CO poisoning if history and symptoms are consistent with this phenomenon. Differential diagnoses cor* *re* *spond* *ing to the symptoms must be considered.*

- Yes: 11, no: 0, abstention: 0
- Strength of consensus: 100% (strong consensus)

The diagnosis of CO poisoning is essentially based on clinical symptoms and suspected or proven exposure [36].

*In the emergency department, the source of exposure should always be determined, especially for patients with CO poisoning that have not been brought in by emergency medical services, in order to identify other poisoned persons and, if necessary, prevent further CO poisoning from as yet unknown sources.*

*In terms of hazard prevention, the fire department should be alerted to check the scene of the accident and undertake the necessary safety measures.*

- Yes: 10, no: 0, abstention: 0
- Strength of consensus: 100% (strong consensus)

*Patients with CO poisoning are not admitted to emergency departments exclusively by ambulance. Patients with CO poisoning also present of their own accord. Therefore, a differential diagnosis of CO poisoning should be considered in emergency departments for patients with nonspecific symptoms such as clouding of consciousness, dizziness, nausea, or vomiting.*

- Yes: 10, no: 0, abstention: 0
- Strength of consensus: 100% (strong consensus)

*Patients in the prehospital or emergency department setting can be left on site as affected individuals or discharged home from the emergency department, respec* *tivel* *y, following CO exposure and a thorough clinical examination that reveals no symptoms, as well as instrument-based diagnostics, after consideration of the individual risk constellation and an evaluation of relevant possible differential diagnoses.*

- Yes: 9, no: 0, abstention: 1
- Strength of consensus: 100% (strong consensus)

### 4.1 Symptoms of acute carbon monoxide poisoning

**How can the severity of CO poisoning be assessed? What are typical and possible early symptoms of acute CO poisoning?**

Acute CO poisoning varies considerably in its clinical presentation. It ranges from mild and nonspecific symptoms such as headache, difficulty concentrating, confusion, visual disturbances, nausea, dizziness, vomiting, abdominal pain, dyspnea, and chest pain to unconsciousness, hypotension, severe acidosis, and acute circulatory failure. 

The most common presenting symptoms include headache, nausea, vomiting, dyspnea, dizziness, and syncope [37], [38], [39].

The brain and heart are especially at-risk organs that also cause symptoms due to their low hypoxia tolerance and high oxygen demand.

The following symptoms may indicate CO poisoning:

- Headache
- Dizziness
- Feeling of weakness
- Nausea, vomiting

### 4.2 Signs of severe carbon monoxide poisoning

The following symptoms may correlate with severe CO poisoning:

- Impaired orientation
- Impaired consciousness
- Seizure
- Angina pectoris
- Cardiac arrhythmia
- Dyspnea, tachypnea
- Pulmonary edema
- ECG changes or abnormal cardiac biomarkers
- Metabolic acidosis
- Extremely high COHb levels (blood gas analysis [BGA] value upon cessation of exposure)

### 4.3 Diagnostic methods

**Which diagnostic methods are needed by emergency medical services, as well as in the context of clinical first aid, in CO poisoning?**

*CO pulse oximetry can be used in the prehospital setting to support a suspected diagnosis. A negative reading, especially if symptoms are present, should not be used to rule out CO poisoning.*

- Yes: 10, no: 0, abstention: 1
- Strength of consensus: 100% (strong consensus)

*To support diagnosis, venous, arterial, or capillary blood sampling for COHb measurement by BGA should be performed in the prehospital setting.*

- Yes: 10, no: 0, abstention: 0
- Strength of consensus: 100% (strong consensus)

A validated spectrophotometric method (BGA) is usually not available in the preclinical setting. For the best possible assessment of the highest COHb value, it is advisable to take a blood sample as early as possible. To this end, it is irrelevant whether a venous, arterial, or capillary blood sample is taken. This blood sample does not need to be stored in a special manner [40].

Normal pulse oximeters are not suitable for distinguishing between COHb and oxyhemoglobin [41], [42]. The use of eight-wavelength pulse oximeters makes detection possible [43], [44]. However, since insufficient accuracy has been reported [45], they are currently not recommended by the American College of Emergency Physicians for the diagnosis of acute CO poisoning [46]. Nevertheless, since the measured COHb level is only one component in the evaluation of the overall clinical symptoms, the guideline group believes that pulsoximetric determination by the emergency services is useful for orientation [47], [48].

COHb levels of at least 3–4% are considered elevated and abnormal [1], [49]. In smokers, COHb levels can be as high as 10% without causing symptoms [36].

## 5 Emergency rescue

### 5.1 Lay responders

**What measures are recommended for lay responders?**

*If signs of a potential hazard involving CO are detected, first responders should be instructed by the emergency dispatch center to observe measures of self-protection, and the information should be relayed to the emergency medical services team.*

- Yes: 8, no: 0, abstention: 0
- Strength of consensus: 100% (strong consensus)

As a colorless, odorless, and tasteless gas, CO is undetectable to the senses and causes nonspecific symptoms when inhaled (see Sect. 4.1). For the first responder, the suspected diagnosis of “CO poisoning” can be difficult to verify. It is important for the first responder to recognize any threat of CO exposure. While observing measures of self-protection, the lay responder should immediately remove the patient from the hazardous area and undertake symptom-oriented first aid measures. If first responders identify signs of a potential hazard from CO, guidance on self-protection from the control center is appropriate.

### 5.2 Emergency responders 

**What action should be taken by emergency responders when CO is detected or suspected in the ambient en** **vi** **ronme** **nt?**

*If CO is detected or suspected in the ambient envi* *ronment, respiratory protection must be used by fire department responders.*

- Yes: 10, no: 0, abstention: 0
- Strength of consensus: 100% (strong consensus)

Between 2012 and 2015, the German Firefighters Association (Deutscher Feuerwehrverband e.V., DFV) [50], the German Fire Protection Association (Vereinigung zur Förderung des Deutschen Brandschutzes e.V., vfdb) [51], and the German Social Accident Insurance (Deutsche Gesetzliche Unfallversicherung e. V., DGUV) (Attachment 1 ) [52], among others, published statements on alerting emergency personnel to ambient air contaminated by CO. Since the recommendations relate to different areas of application, the recommended threshold values differ (Table 2 (Tab. 2)).

The DGUV statement refers to the alerting of emergency services personnel to unexpected CO in ambient air during the performance of routine emergency and patient transportation operations.

**What steps should be taken by emergency services personnel in the event of unexpected CO in ambient air?**

*When CO warning devices are used by emergency services, a multi-stage approach ought to be taken depending on the concentration indicated.*

- Yes: 10, no: 0, abstention: 0
- Strength of consensus: 100% (strong consensus)

If CO warning devices unexpectedly detect CO in ambient air during the course of routine emergency and ambulance operations, the emergency services team must leave the hazardous area as quickly as possible. The rescue of patients must be carried out with due regard to self-protection and regional instructions for action.

By establishing multi-level warning thresholds, it is possible to ensure that health hazards are unlikely and that emergency personnel are able to perform at their full capacity, while at the same time ensuring that the best possible patient care remains possible.

Table 2 (Tab. 2) provides an overview of appropriate responses and measures according to the “Recommendation of the DGUV on the use of carbon monoxide warning devices by fire departments and emergency services” (Attachment 1 ) [52].

## 6 Prehospital management

**What measures are recommended for healthcare professionals? At what point is oxygen breathing indicated in CO poisoning?**

*If CO poisoning is suspected, 100% oxygen or ventilation should be started immediately.*

- Yes: 10, no: 0, abstention: 0
- Strength of consensus: 100% (strong consensus)

**How should oxygen be administered?**

*Oxygen administration is the most important measure of prehospital care for CO poisoning.*

*Regardless of the oxygen saturation (SpO2), oxygen should be administered immediately at the highest possible concentration.*

*–* *Mask continuous positive airway pressure (CPAP) (non-invasive ventilation, NIV) or*

*– Demand valve or*

*– Constant dosing (high-flow) via tight-fitting mask with reservoir bag or*

*– Invasively using appropriate airway protection if protec* *tive* *reflexes are inadequate.*

- Yes: 8, no: 0, abstention: 0
- Strength of consensus: 100% (strong consensus)

Spontaneous breathing with mask CPAP (NIV) is an effective mode of oxygen administration in prehospital management for the elimination of CO. There is strong evidence both in case reports [53], [54] and in several prospective studies that CPAP therapy at 5–12 mbar significantly shortens the half-life of COHb [55], [56], [57].

*Prehospital monitoring should include pulse oximetry, respiratory rate, ECG, and noninvasive blood pressure (NIBP).*

- Yes: 11, no: 0, abstention: 0
- Strength of consensus: 100% (strong consensus)

## 7 Hospitalization

*Procedure for deciding on hospitalization for patients with acute CO exposure:*

1. *Symptomatic patients: hospitalization always recommended!*
2. *Asymptomatic patients:*

*– Up to 5% COHb (in smokers: 10%): hospitalization not recommended*

*– Hospitalization ought to be considered or offered to pregnant women and children*

- Yes: 10, no: 0, abstention: 0
- Strength of consensus: 100% (strong consensus)

During transport, oxygen breathing or ventilation that has already been started must be continued.

In close consultation with the responsible control center, the emergency physician ought to decide on a maximum transport time of 30–40 min and, as such, usually also on the nearest suitable hospital.

At the receiving hospital, a decision should be made promptly regarding the need for secondary transfer to an HBOT center, and the center contacted if necessary (see Sect. 9).

## 8 Initial in-hospital care

**Which investigative procedures are valid for the diagnosis and follow-up of CO poisoning?**

**Which diagnostic methods are required for CO poisoning as part of initial in-hospital care?**

**Which other tests are recommended after a patient arrives in the emergency department?**

*To detect elevated COHb levels and determine pH and lactate, BGA should be performed in all patients with suspected CO poisoning.*

- Yes: 10, no: 0, abstention: 0
- Strength of consensus: 100% (strong consensus)

*A neurological examination (including, for example, the mini-mental state examination [MMSE]) should be performed in patients with clinical symptoms. If there is sufficient suspicion of a relevant differential di* *ag* *no* *sis, further specific examinations ought to be performed.*

- Yes: 10, no: 0, abstention: 0
- Strength of consensus: 100% (strong consensus)

*A determination of biomarkers of myocardial injury, such as creatine kinase (CK), CK-muscle and brain (CK-MB), and troponin, as well as a 12-lead ECG should be performed in all patients with suspected CO poisoning, especially those with cardiac symptoms and history. If there is sufficient suspicion of relevant differential diagnoses, further organ-specific diagnostics should be performed.*

- Yes: 10, no: 0, abstention: 0
- Strength of consensus: 100% (strong consensus)

*If there is evidence of concomitant poisoning or suicidal CO poisoning, toxicological screening (e.g., blood ethanol level, drug screening in blood or urine) should be performed.*

- Yes: 10, no: 0, abstention: 0
- Strength of consensus: 100% (strong consensus)

Since the symptoms of CO poisoning are nonspecific, may be many and varied, and, in some cases, no clear source of CO can be identified in the medical history, other differential diagnoses should be included and investigated at an early stage with a low threshold and under continued oxygen therapy, especially in the case of pronounced cardiac or neurological symptoms and lack of clinical improvement despite treatment. This can include various instrument-based and invasive examinations such as intracranial imaging, echocardiography, cardiac catheterization, etc.

**Lactate, pH:** Metabolic acidosis with elevated lactate levels may manifest as a sign of tissue hypoxia secondary to CO poisoning. The determination of pH and lactate is helpful to estimate the extent of the patient’s impairment and the severity of hypoxia. For evaluation of acid-base status, arterial measurement is preferable. Using a database analysis of 1,505 patients, Hampson et al. showed that initial pH<7.2 increased mortality to as much as 50%, independent of COHb level [58]. Nevertheless, whether there is a correlation between severity and lactate level in CO poisoning alone is unclear [59]. Furthermore, particularly in the case of smoke inhalation, one must in principle assume combined poisoning. Following smoke inhalation and in the absence of clinical improvement despite adequate oxygenation, combined with marked acidosis (pH<7.2) or high lactate levels >10 mmol/l, cyanide poisoning is highly likely.

**Neurological examination:** Obtaining an initial neurological baseline status is useful for detecting important differential diagnoses and assessing DNS.

**ECG, troponin, CK, CK-MB:** ECG and determination of cardiac biomarkers are useful in all patients with suspected CO poisoning [46]; patients with CO poisoning are at increased risk for cardiac damage [29], [30], [60].

**Toxicology:** In cases of suspected intentional or suicidal CO poisoning, Weaver et al. recommend additional toxicological screening, including blood ethanol levels [35]. In one study, 183/426 patients with intentional CO poisoning were found to have additional intoxication, primarily with alcohol [61].

**S100B:** There is evidence for a possible correlation between the neuronal marker S100B level in serum and the severity of CO poisoning [62], [63], [64], [65], [66]. At present, the data appear insufficient for a general recommendation.

**Monitoring clinical course**

*As long as patients are symptomatic, clinical monitoring commensurate with disease severity should be performed.*

- Yes: 11, no: 0, abstention: 0
- Strength of consensus: 100% (strong consensus)

*Patients’ clinical symptoms do not correlate with COHb clearance from the blood. COHb monitoring alone is unsuitable for treatment management.*

- Yes: 11, no: 0, abstention: 0
- Strength of consensus: 100% (strong consensus)

*Particularly in the absence of improvement despite treatment, other potential differential diagnoses ought to be reevaluated.*

- Yes: 11, no: 0, abstention: 0
- Strength of consensus: 100% (strong consensus)

The primary goal of treatment is to eliminate CO from the body in order to prevent acute and long-term sequelae. Treatment must be continued until the COHb level has dropped to normal values (<3%) and the 36 is symptom-free [36]. This is typically achieved after a maximum of five physiological half-lives for COHb at 100% oxygen breathing (approximately 375 min).

Nevertheless, based on the literature review and the consensus discussion conducted by the guideline group, there is no clear correlation between the level of COHb, COHb clearance, and the patient’s clinical symptoms [36], [67], [68].

From a toxicological point of view, the COHb level after clinical symptoms nevertheless remains a parameter by which acute toxicity can be shown.

The further intensive care treatment of patients with CO poisoning does not differ from the otherwise applicable principles and recommendations for intensive care treatment.

### 8.1 Care of pregnant women

Randomized trials in pregnant women are lacking; recommendations are based on theoretical [69] and animal experimental work [70], as well as on analyses from trauma care [71].

In the fetal system, both saturation and elimination appear to be slowed. Especially in the case of prolonged exposure, fetal COHb levels may even exceed maternal levels [72]. In one case report, fetal autopsy showed a COHb level of 61%, although the mother had a COHb level of 7% after only 1 h of oxygen treatment. Thus, some authors consider pregnancy to be a strict indication for HBOT [73], especially when neurological symptoms, signs of fetal stress, syncope, or a high COHb level are present [14].

### 8.2 Care of children and adolescents

**Are there differences between the treatment of children and adults?**

Special vote of the GNPI and GfKT*:

*The evidence-based data on the therapeutic effectiveness of HBOT of CO poisoning in children is insufficient. An extrapolation of the experience in adults, for which there is also scant evidence, is not permissible in a straightforward manner due to particular pediatric features. In view of this, the anticipated burden and hazard posed by transport to an HBOT facility, as well as during the delivery of this therapy, carries more weight. Therefore, HBOT for CO poisoning in children can only be considered in specific individual cases. The earliest possible continuous availability of pediatric intensive care expertise in the care of these patients is indispensable.*

*The GfKT was a non-voting medical society that did not actively participate in the guideline group. The approved draft of the guideline text was submitted to this medical society for comment. This guideline was endorsed subject to the declaration of this special vote.

The symptoms of acute CO poisoning in school-aged children and adolescents are comparable to those in adults, ranging from headache, nausea, and vomiting to neurological symptoms and coma. While syncope is more common in children than in adults, ECG changes or signs of cardiac ischemia are far less common.

In contrast, the initial symptoms of CO poisoning in childhood are often similar to those of a viral illness and may include impaired vigilance, seizures, and vomiting, albeit in the absence of fever. In infants and young children, diarrhea is not uncommon; on the other hand, poor drinking behavior or irritability may be the only signs. 

Compared to adults, children may show symptoms at lower COHb levels [74], but there is also no reliable correlation of symptoms with COHb concentration. Symptoms have been described at 3% COHb, whereas a newborn was asymptomatic at a level of 22% [75]. 

From experience, children appear to become symptomatic more rapidly, but also show faster recovery and overall lower mortality compared to adults. One study showed a shorter COHb half-life for pediatric compared to adult patients [76].

Newborns and fetuses (in maternal CO poisoning) may have increased sensitivity to CO, since CO accumulates in fetal hemoglobin (HbF) (with levels sometimes higher than in maternal blood) and elimination is slowed. Accordingly, fetuses may be more affected than their mothers (see Sect. 8.1). The toxicity-promoting effect of the higher CO affinity of HbF also affects infants, since HbF, starting from perinatal levels of about 60%–85%, does not drop to near 0.2%–12% until 12 months of age.

DNS are probably less frequent in children compared to adults. The quality of data is insufficient: the available publications show a small number of cases and heterogeneity in all aspects, including the definition of DNS and the methodology of neurological evaluation.

A retrospective study of 106 pediatric patients treated with normobaric oxygen therapy (NBOT) showed persistent neurological symptoms that may have been sequelae of CO poisoning in only three children [77].

The diagnostic and therapeutic procedure in children is similar to that in adult patients, but clinical and prognostic features lead to a differentiation in the grades of recommendation. In general, the clinical symptoms should determine the indication for therapy and not only the COHb value. 

The earliest possible administration of 100% high-flow oxygen by mask or via high-flow nasal cannulae (HFNC) is mandatory; intubated children should be ventilated with a fraction of inspired oxygen (FiO2) of 1.0 until they are symptom-free and their COHb value has dropped to normal levels (<3%). Admission to a pediatric hospital with intensive care facilities should take place.

The effectiveness of HBOT in pediatric CO poisoning is unclear. Furthermore, additional stress and risks arise as a result of the required transport and prolonged stay outside the ICU, meaning that HBOT in pediatric patients with CO poisoning only ought to be performed under special conditions with a specific indication.

The available prospective randomized trials of HBOT versus NBOT for CO poisoning have been conducted in adults, with some including adolescents older than 15 years [78] or 16 years [18], [79]. However, a precise figure for the number of pediatric patients is lacking.

A larger retrospective study from Taiwan failed to demonstrate a significant difference in mortality between children with or without HBOT [80]; however, patients treated with HBOT showed more severe initial symptoms.

Another larger, likewise retrospective series found better neurological outcomes with HBOT, although the indication for HBOT, and thus the comparability of the HBOT to NBOT cohort, remains unclear [33].

Overall, there is no methodologically sufficient study in pediatric patients to demonstrate or exclude a therapeutic effect for HBOT. Guidelines from other countries do not specifically comment on HBOT in children. Therefore, the burden and potential complications or side effects of HBOT play a significant role in weighing up whether HBOT ought to be performed.

Complications of HBOT described in infancy include seizures, pulmonary barotrauma, and hypothermia. When considering HBOT in the neonatal or preterm period, particular attention must be paid to the possibility of oxygen toxicity leading to oxygen-induced retinopathy (in premature infants). In addition, pulmonary malformations such as congenital lobar emphysema lead to a significantly increased risk of pneumothorax. These ought to be excluded by thoracic imaging before performing HBOT. 

HBOT may be considered in individual cases where severe disturbance of consciousness caused by CO poisoning persists for several hours despite administration of oxygen, stabilization of vital signs, and in the absence of any other plausible explanation for the impaired vigilance (e.g., traumatic brain injury, cyanide poisoning). In addition, it is essential for the initiation of HBOT in the pediatric patient that transport and treatment do not jeopardize the patient’s stabilization. Therefore, initial care always ought to be provided on a pediatric intensive care unit and the patient should be stable and fit to be transported without major risks.

For the care of seriously ill children and adolescents during HBOT, special experience in the intensive medical care of children must always be available.

The statement of the German Society for Neonatology and Pediatric Intensive Care Medicine (GNPI) should be seen as a basic prerequisite for the clinical treatment of critically ill children and adolescents [81].

The emergency care of infants with severe CO poisoning is generally provided in accordance with this statement of the GNPI.

## 9 Hyperbaric oxygen therapy (HBOT)

**When is hyperbaric therapy indicated?**

*In the case of signs of severe CO poisoning (including continued impaired consciousness, metabolic acidosis, respiratory insufficiency, and/or cardiac ischemia), as well as during pregnancy, HBOT ought to be administered in adults (aged 18 years and over).*

- Yes: 7, no: 2*, abstention: 1
- Strength of consensus: 78 (consensus)

*The DGINA and DGIIN did not approve this recommendation. The special vote of the DGINA and DGIIN, as well as of the DGP and GfKT, which are not entitled to vote here, should be seen in this context (see below). The mandate holder of the DGAI abstained due to conflicts of interest.

Evidence on the benefit of hyperbaric oxygen is low due to heterogeneous studies [82], [83], [84]. The most recent meta-analysis by Wang et al. included seven randomized controlled trials (2023 patients) with neurological deficit as the endpoint [85].

In the search and selection of evidence sources for this guideline, the Clinical Policy published by the American College of Emergency Physicians (ACEP) was rated as being of high quality (including direct topic relevance, representative author group, systematic evidence-based literature search, defined development and, where necessary, consensus process, final expert review, and accessibility to the expert community) and consequently selected [46].

In line with the reasoning of the ACEP and after extensive discussion within the guideline group, considerable importance was attached to the different HBOT treatment regimens (treatment pressures, treatment times) and the time-to-treatment factor in the available randomized trials.

The potential benefits of HBOT have been demonstrated with therapy within 6 h and therapy regimens using treatment pressures of 2.5–3 bar [46], [86]. For this reason, the treatment recommendations in this guideline are explicitly in line with the study by Weaver et al. [35]. The time factor has also been presented by Liao et al. [87].

The incidence of barotrauma in HBOT is reported to be approximately 0.72%, and other complications (hypoglycemia, oxygen toxicity, dizziness, anxiety, shortness of breath, chest tightness) to be 0.5–1.5% [88]. In a study by Eichhorn et al. [89], hyperbaric treatment of 476 patients with CO poisoning in Germany was discontinued in six patients due to problems in middle ear pressure equalization. Damage to the tympanic membrane was not observed. Circulatory problems occurred in four cases during the first HBOT session without further sequelae. No health- or life-threatening events occurred.

Special vote of the DGINA and DGIIN, as well as of the DGP* and GfKT*:

*Patients (children, adults, and pregnant women) with CO poisoning ought to receive HBOT* *or* *NBOT at high flow rates.*

*Due to the lack of evidence, it remains unclear to the DGINA, DGIIN, DGP, and GfKT whether HBOT offers an advantage over NBOT in improving neurocognitive treatment outcomes in the long term. Due to the risks and potential complications of HBOT, the decision to use this therapy remains a case-by-case decision.*

*The DGP and GfKT were non-voting medical societies that did not actively participate in the guideline group. The approved draft of the guideline text was submitted to these medical societies for comment. This guideline was endorsed subject to the declaration of this special vote.

**Within what time frame should patients be transferred for hyperbaric therapy?**

*HBOT should be initiated within 6 h.*

- Yes: 9, no: 0, abstention: 1
- Strength of consensus: 100% (strong consensus)

HBOT is not recommended after more than 24 h [90].

**What hyperbaric treatment schedules should be used?**

*HBOT should be performed three times within 24 h. The initial HBOT session should correspond to the therapy schedule (TS) 300/90 (according to Boerema schedule). A second and third HBOT session should be performed at a treatment pressure greater than/equal to 2.4 bar (TS 240/90).*

- Yes: 7, no: 0, abstention: 3
- Strength of consensus: 100% (strong consensus)

For the therapeutic success of HBOT, a sufficiently high initial total pressure is presumably required for a sufficient duration. On the other hand, both the total pressure and the duration of therapy must be limited to keep the risk of possible side effects low. 

**Figure 1:**
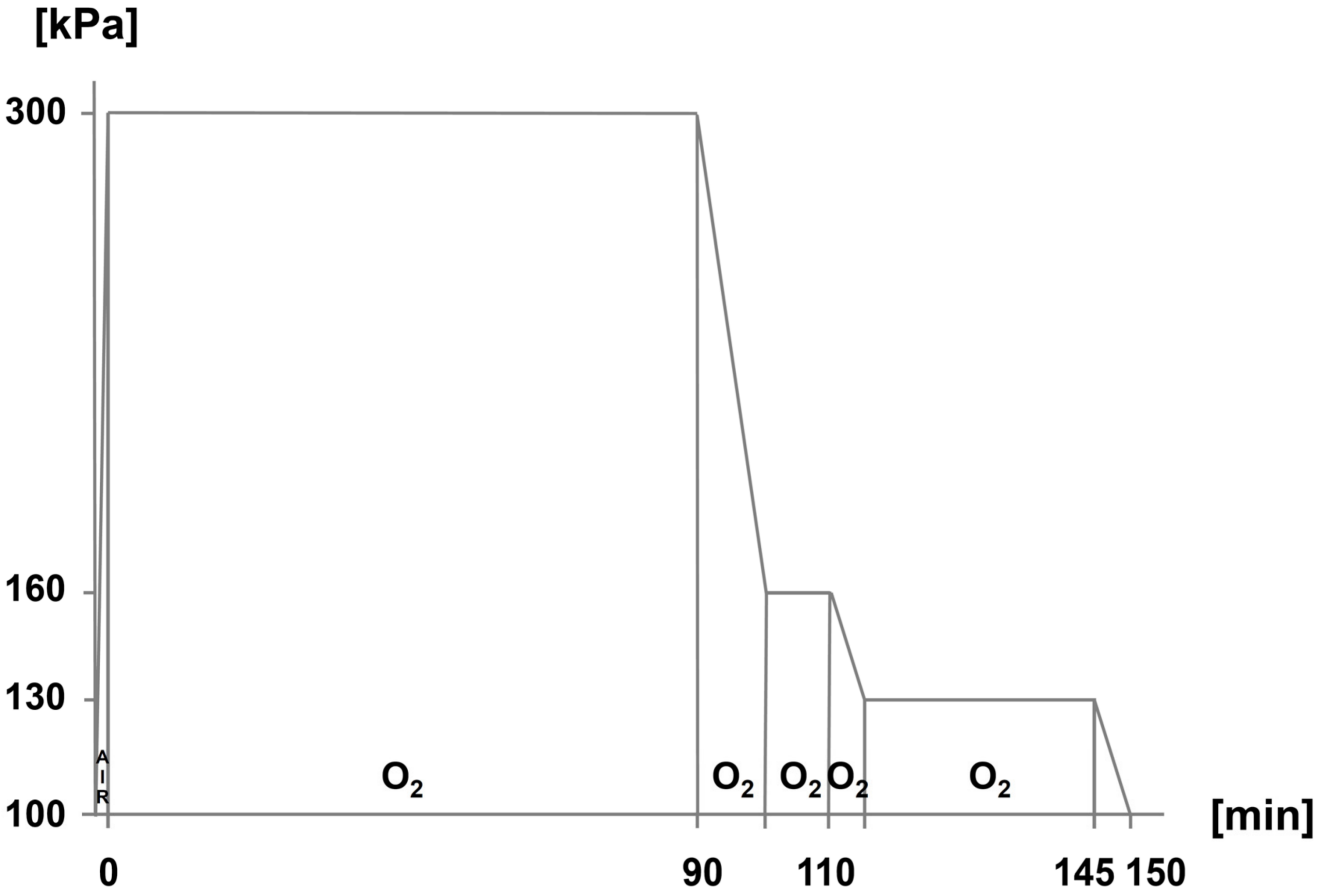
Hyperbaric oxygen therapy: therapy schedule 300 kPa for 90 min oxygen breathing (TS 300/90)

**Figure 2:**
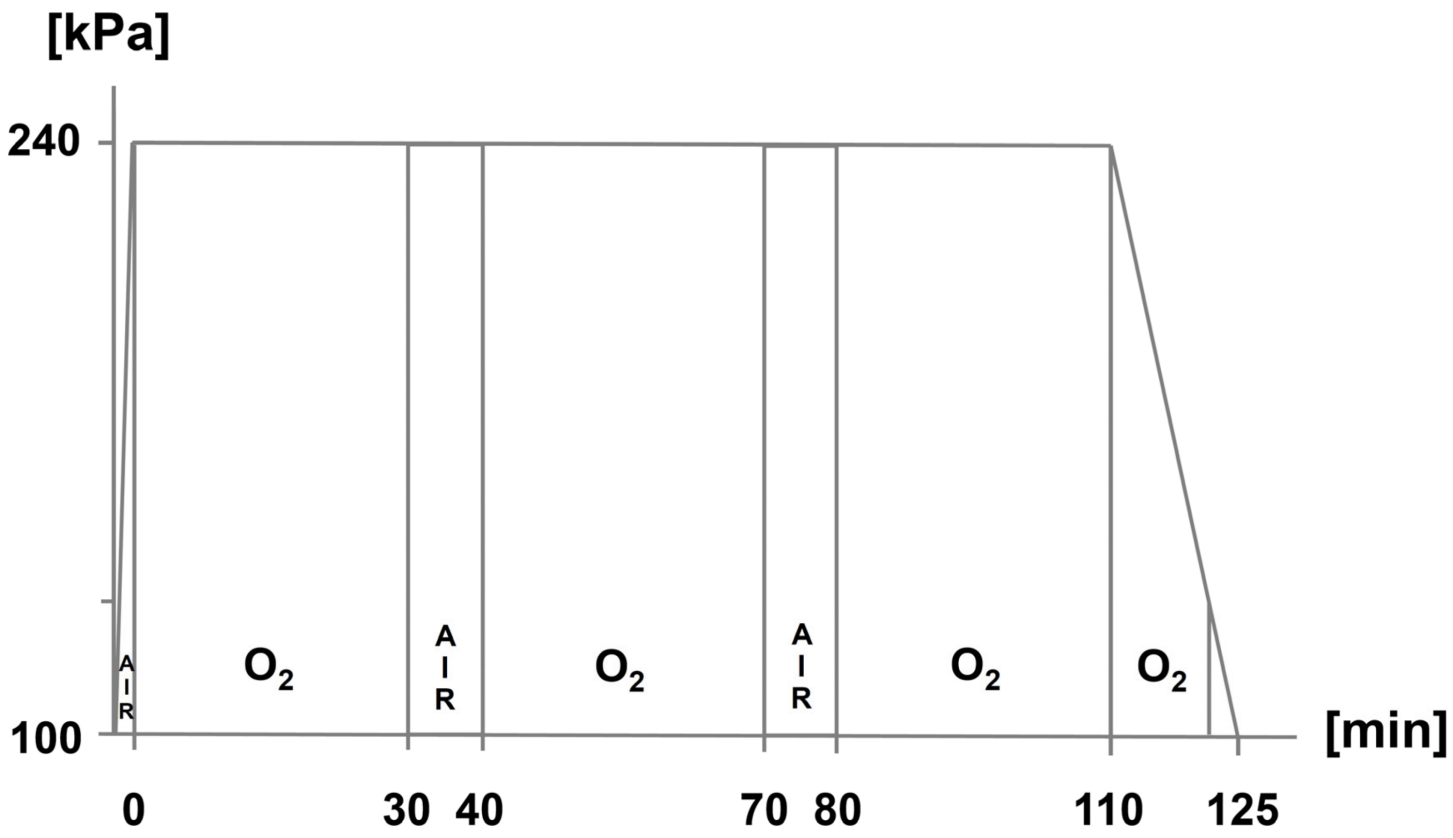
Hyperbaric oxygen therapy: therapy schedule 240 kPa for a total of 90 min of oxygen breathing (TS 240/90)

In the European literature, the TS 300/90 (so-called Boerema schedule, 300 kPa total pressure for 90 min with oxygen breathing followed by gradual decompression) and TS 240/90 (240 kPa total pressure for 90 min with oxygen breathing) are described (Figure 1 (Fig. 1), Figure 2 (Fig. 2)). These therapy schedules correspond to the quality standards for HBOT most recently published by the German Society for Diving and Hyperbaric Medicine (Gesellschaft für Tauch- und Überdruckmedizin, GTÜM e.V.) [91].

**What pressure chamber equipment is required for pressure chamber treatment?**

*When taking on the treatment of a patient (potentially) in need of intensive care, their care should be provided according to intensive care standards (this includes, among other things, a ventilator and monitoring equipment approved for operation in an HBOT chamber) before, during, and after pressure chamber treatment.*

- Yes: 9, no: 0, abstention: 1
- Strength of consensus: 100% (strong consensus)

Furthermore, the relevant regulations in the DGUV Information 207-001 “Working safely with therapeutic pressure chambers” must be observed [92].

**What personnel qualifications are required for pressure chamber treatment?**

Qualification of on-site personnel during therapeutic pressure chamber treatments [93]:

I. For pressure chamber treatment of inpatients or outpatients not requiring intensive care and pressure chamber treatment of emergencies.

- One physician with a “Hyperbaric Medicine Physician” diploma (GTÜM e.V.) and “Hyperbaric Operator” diploma (GTÜM e.V., VDD e.V.) and
- One “Hyperbaric Medical Assistant” (GTÜM e.V., VDD e.V.) or one further physician and
- One “Hyperbaric Operator” (GTÜM e.V., VDD e.V.)

If the physician and assistant are in the pressure chamber at the same time, an additional person (with a pressure chamber operator qualification) is required outside the pressure chamber in accordance with DGUV-I 207-001 (a total of four persons: two inside the pressure chamber, two outside the pressure chamber).

II. For pressure chamber treatment of patients requiring intensive care

- One physician with a “Hyperbaric Medicine Physician” diploma (GTÜM e.V.) and “Hyperbaric Operator” diploma (GTÜM e. V., VDD e.V.) and
- One “Hyperbaric Nurse” (GTÜM – VDD e.V.) or one additional physician with at least 1 year of further training in anesthesia or intensive care medicine and
- One “Hyperbaric Operator” (GTÜM e. V., VDD e.V.)

If the physician and assistant are in the pressure chamber at the same time, an additional person (with a pressure chamber operator qualification) is required outside the pressure chamber in accordance with DGUV-I 207-001 (a total of four persons: two inside the pressure chamber, two outside the pressure chamber).

## 10 Rehabilitation/late sequelae

**What are typical and possible late symptoms of acute CO poisoning?**

*All patients with CO poisoning should be educated about the risk of DNS, symptoms, and time of onset. If DNS is suspected, the patient should present to a neurologist.*

- Yes: 10, no: 0, abstention: 0
- Strength of consensus: 100% (strong consensus)

*Interval cardiac follow-up ought to be considered for signs of cardiac injury in the setting of acute CO poi* *son* *ing in order to detect long-term cardiac damage.*

- Yes: 10, no: 0, abstention: 0
- Strength of consensus: 100% (strong consensus)

A risk of DNS following CO poisoning has been described [35]. It is unclear whether these are specifically new symptoms or ongoing symptoms. Onset is possible in immediate temporal relation to exposure or after a symptom-free interval. More commonly, the occurrence of DNS is reported within the first month after CO poisoning. In some cases, DNS onset also seems to be possible with a significant delay within the first year following CO poisoning [10], [22], [94], [95]. The probability of occurrence is up to 50% [96].

There is an increased risk of DNS after initial loss of consciousness [94], [2], [97]. Other risk factors for the development of late neuronal damage are considered to include age >36 years (odds ratio [OR]: 2.6 [1.3–4.9]) and exposure time exceeding 24 h (OR: 2.0 [1.0–3.8]; p=0.046) [98].

Symptoms of DNS include motor dysfunction, Parkinsonism, behavioral changes, memory impairment, headache, dizziness, depression, and the development of dementia.

In addition to neurological sequelae after CO poisoning, further clinical studies describe the occurrence of diabetes mellitus, cardiovascular events, and increased long-term mortality [19], [29], [30], [99].

Patients should be evaluated for cognitive sequelae 4–6 weeks after CO poisoning [85]. Cardiac follow-up appears reasonable if there are signs of primary cardiac injury.

## 11 Quality assurance

Guidelines are intended to represent a good basis of information, provide orientation, and, as decision-making aids, promote the transfer of the best available evidence from clinical trials and the professional consensus of experts into everyday care [100].

Guidelines are also able to support concrete decision-making and action-taking processes, especially in rare emergencies.

To evaluate the application of this guideline and review its implementation, quality indicators are to be developed and recorded. Taking into account the course of care, parameters are to be defined that evaluate process, structural, and, if necessary, outcome quality.

In the following, the guideline group has drafted suggestions for indicators and metrics that will need to be further developed and reviewed in their application following publication of this S2k guideline.

In principle, administrative routine data, for example from the data sets of the German Interdisciplinary Association for Intensive Care and Emergency Medicine (DIVI) emergency physician protocol and emergency admission register [101], as well as, if necessary, data from a national register for HBOT to be established in Germany should be used for this purpose.

The data definition in this guideline will be integrated into the DIVI emergency department protocol data set.

### 11.1 Preclinical quality indicators

**Figure 3:**
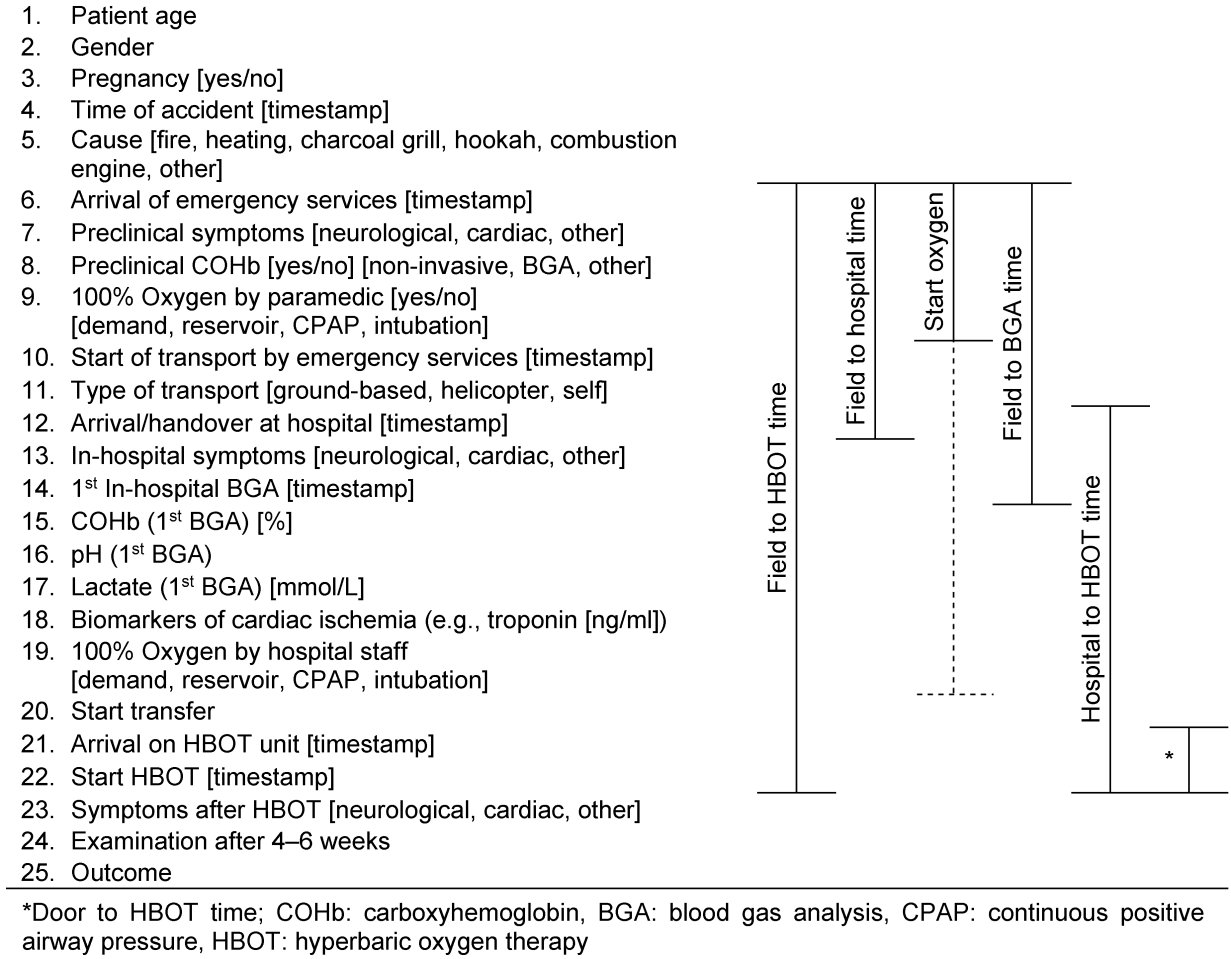
Parameters of the course of care with quality of process indicators (modified from [102])

Taking into account the course of care, parameters were described and further performance indicators formulated (Figure 3 (Fig. 3)).

1. 100% Oxygen breathing in the case of suspected CO poisoning
  → “Start oxygen”
  [Time interval from diagnosis to start of oxygen therapy].
2. The diagnosis of CO poisoning requires clinical symptoms and proven or probable exposure to CO.
  To support the diagnosis, a preclinical venous or capillary blood sample should be taken for COHb determination by BGA.
  → “Field to BGA time”
  [Time interval from arrival of emergency services to first BGA].

### 11.2 Clinical quality indicators

Treatment in the emergency department begins with the initial medical assessment and ends with the transfer or discharge of a patient from the emergency department.

If a patient is diagnosed with CO poisoning:

1. Symptoms should be documented at the time of admission, progress documented during emergency room treatment, and symptoms documented at the time of discharge/transfer.
  → “Documentation”
  [Documentation of symptoms].
2. Oxygen should be started or continued with the highest possible concentration without delay.
  → “Start oxygen”
  [Time interval from diagnosis to start of oxygen therapy].
3. HBOT should be administered in the case of signs of severe CO poisoning (including ongoing impaired consciousness, metabolic acidosis, respiratory insufficiency, and/or cardiac ischemia), as well as in adult pregnancy (18 years of age).
  → “Field to HBOT time”
  → “Hospital to HBOT time”
  [Time intervals to start of HBOT].

From an emergency department perspective, all BGAs should be checked for elevated COHb levels.

1. Elevated COHb values (>3%) should be noted in the emergency department report, whether or not they correspond to CO poisoning when all findings and clinical symptoms are considered together. The percentage of unnoted COHb values >3% should be less than 1%.

### 11.3 Post-inpatient quality indicators

If a patient with CO poisoning and neurological or cardiological pathologies is transferred, the transfer report should indicate the need for further post-inpatient follow-up.

1. Patients should be assessed for cognitive sequelae 4–6 weeks after CO poisoning.
  → “Outcome”
  [Neurological examination (e.g., MMS test)]
2. Cardiological follow-up appears judicious if there is evidence of primary cardiac injury.
  → “Outcome”

### 11.4 Updating procedure

The use and implementation of the guideline should be evaluated prior to updating.

## Notes

### Guideline report

The methodological approach to the development of the guideline and, in particular, the management of potential conflicts of interest is presented in the guideline report. This is freely available online, e.g., on the website of the Association of the Scientific Medical Societies in Germany (AWMF) [102].

### Competing interests

See Attachment 2

## Supplementary Material

Empfehlung der DGUV für den Einsatz von Kohlenmonoxidwarngeräten bei Feuerwehren und Hilfsorganisationen

Declaration of interests and management of conflicts of interest

## Figures and Tables

**Table 1:**
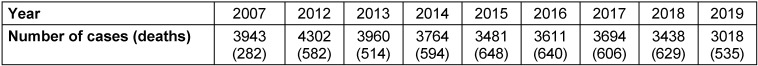
Number of cases and deaths of carbon monoxide poisoning (diagnosis ICD 10) according to German Federal Health Reporting

**Table 2:**
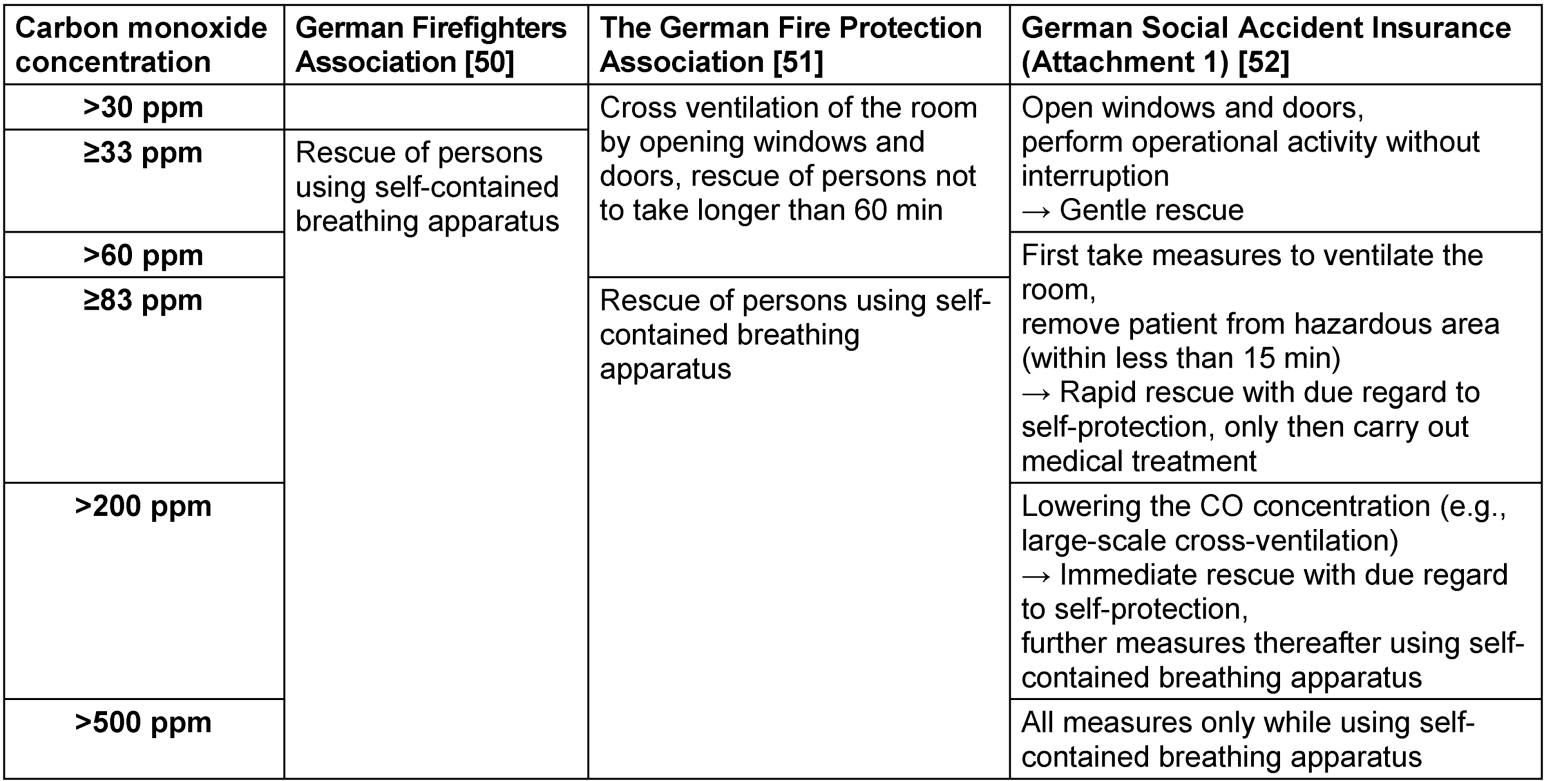
Comparison of recommendations for the operational response of responders to unexpected exposure to carbon monoxide
