# Supplementary material for: S2k guideline diagnosis and treatment of carbon monoxide poisoning
Source: Ger Med Sci. 2021 Nov 4;19:Doc13. doi: 10.3205/000300 (PMC8607608; doi:10.3205/000300)
Supplement: Empfehlung der DGUV für den Einsatz von Kohlenmonoxidwarngeräten bei Feuerwehren und Hilfsorganisationen [file GMS-19-13-s-001.pdf]

Empfehlung der DGUV für den Einsatz von Kohlenmonoxidwarngeräten bei Feuerwehren und Hilfsorganisationen [52]

kommmitmensch
Sicher. Gesund. Miteinander.

DGUV
Fachbereich Feuerwehren
Hilfeleistungen Brandschutz

Fachbereich AKTUELL

FBFHB-021

Einsatz von Kohlenmonoxidwarngeräten bei Feuerwehren und Hilfeleistungsorganisationen

Sachgebiet Feuerwehren und Hilfeleistungsorganisationen   Stand: 14.09.2020

Im Zusammenhang mit Einsätzen der Feuerwehr und des Rettungsdienstes kann es seit jeher zur unbemerkten Exposition der Einsatzkräfte mit Kohlenmonoxid (CO) kommen. Neben klassischen Brandeinsätzen gewinnen Schadensereignisse ohne initiales Brandgeschehen, wie z. B. unbeabsichtigte CO-Freisetzungen durch defekte Feuerstätten oder vorsätzlich herbeigeführte CO-Freisetzungen in suizidaler Absicht, immer mehr an Bedeutung (weitere Informationen siehe auch DFV-Fachinformation 04/2012 Rahmenempfehlung zu Einsätzen bei Verdacht auf einen CO-Notfall innerhalb von Räumen[1]) bzw. Gefahrstoffdatenbank GESTIS[2] der DGUV.

Im Zuge der Einsatzvorbereitung ist es daher sinnvoll, anhand einer Gefährdungsbeurteilung (z. B. gemäß [5]) zu prüfen, in welchem Umfang im Feuerwehr- und Rettungsdiensteinsatz CO-Warngeräte mitgeführt werden sollen.

Führen Feuerwehren oder Rettungsdienste als Ergebnis ihrer Gefährdungsbeurteilung CO-Warngeräte mit, sind Maßnahmen je nach Expositionshöhe zu ergreifen. Aus Sicht des Sachgebietes Feuerwehren und Hilfeleistungsorganisationen (SG FwH) und des Instituts für Prävention und Arbeitsmedizin der DGUV (IPA) sind die in **Tabelle 1** beschriebenen Warnschwellen und Verhaltensweisen zu empfehlen.

Hierfür wurde zugrunde gelegt, dass:

- die exponierten Einsatzkräfte gesund sind bzw. keine Schwangerschaft vorliegt, da eine Fruchtschädigung bereits bei geringer CO-Exposition nicht ausgeschlossen werden kann. Insbesondere Personen mit koronaren Herzerkrankungen (KHK) können bei körperlicher Belastung deutlich empfindlicher auf eine CO-Exposition reagieren, als gesunde Menschen. *Anmerkung:* Eine KHK kann vorliegen, auch wenn die Einsatzkraft sich subjektiv gesund fühlt. Darüber hinaus gibt es momentan keine nichtinvasive Untersuchungsmethode, um eine KHK sicher auszuschließen.
- bei potenziell exponierten Einsatzkräften und Patienten eine umgehende Blutentnahme für die CO-Hb-Bestimmung in einer Klinik oder eine präklinische Schnelldiagnostik mit CO-Hb-Pulsoxymeter erfolgt,
- die Messgeräte von den Einsatzkräften ständig personennah getragen werden und ein ausreichender Luftzutritt zum CO-Warngerät gewährleistet ist (siehe **Abbildung 1**),

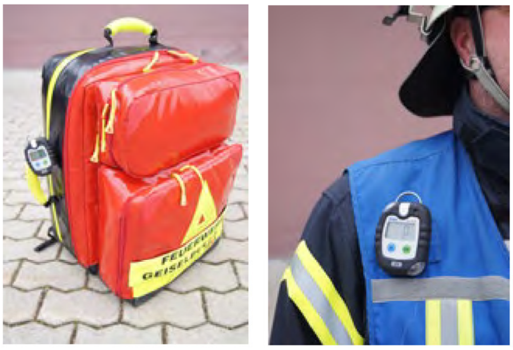

Abb. 1

- die Pflege und Wartung der Geräte gemäß den Herstellervorgaben bzw. dem FBFHB-020 des SG FwH der DGUV erfolgt,
- die Verweildauer der Einsatzkräfte im möglichen Gefahrenbereich nicht mehr als 30 Minuten beträgt,

1 http://www.feuerwehrverband.de/fileadmin/Inhalt/FACHARBEIT/FB6_ELU/DFV-Fachempfehlung_Einsatzstrategien_CO-Notfall.pdf
2 https://www.dguv.de/ifa/gestis/gestis-stoffdatenbank/index.jsp

Attachment 1 to: Jüttner B, Busch HJ, Callies A, Dormann H, Janisch T, Kaiser G, Körner-Göbel H, Kluba K, Kluge S, Leidel BA, Müller O, Naser J, Pohl C, Reiter K, Schneider D, Staps E, Welslau W, Wißuwa H, Wöbker G, Muche-Borowski C. S2k guideline diagnosis and treatment of carbon monoxide poisoning. GMS Ger Med Sci. 2021;19:Doc13. DOI: 10.3205/000300, URN: urn:nbn:de:0183-0003009

- die Einsatzkräfte in Räumlichkeiten tätig werden, in denen mit „haushaltsüblichen“ potenziellen CO-Quellen (z. B. Heizgeräte, Feuerstätten, Verbrennungsmotoren) zu rechnen ist,
- bei Gefahrstofflagen im industriellen Maßstab bzw. gemäß FwDV 500 „Einheiten im ABC-Einsatz“ im Gefahrenbereich von vornherein Einsatzkräfte mit mindestens umluftunabhängigem Atemschutz eingesetzt werden.

!

Dieses FB AKTUELL ersetzt in keinem Fall die eigene, örtliche Gefährdungsbeurteilung sowie evtl. daraus resultierende taktische und strategische Überlegungen bzw. Standardeinsatzregeln/Dienstanweisungen. Ob die Inhalte der Tabelle 1 im eigenen Zuständigkeitsbereich vollständig genutzt oder individuell angepasst werden (z. B. durch das Weglassen einer Warnschwelle) muss mit Hilfe einer Gefährdungsbeurteilung im Rahmen der Einsatzvorbereitung entschieden werden.

Evtl. vorhandene, landesspezifische Regelungen sind vorrangig zu beachten.

CO-Konz.		Maßnahmen/Verhalten
> 30 ppm	Aufmerksamkeitsschwelle	**Achtung: CO vorhanden!**<br>• Fenster und Türen öffnen<br>• Einsatztätigkeit ohne Unterbrechung durchführen → schonende Rettung<br>• CO-Quelle identifizieren und weitere Freisetzung unterbinden, falls das ohne Eigengefährdung möglich ist.<br>• Wenn die Quelle nicht zu ermitteln bzw. abzustellen ist, Fachkräfte (je nach Lage z. B. Feuerwehr, Störungsdienst, Schornsteinfeger) informieren.
> 60 ppm	Gefährdungsschwelle	**Achtung: CO in erhöhter Konzentration vorhanden!**<br>• Zuerst Maßnahmen zur Belüftung des Raums ergreifen!<br>• Wenn effektive Belüftung nicht möglich ist, Patient aus dem Gefahrenbereich bringen (dabei Aufenthaltsdauer im Gefahrenbereich minimieren, Richtwert: unter 15 min)<br>→ schnelle Rettung unter Beachtung des Eigenschutzes.<br>• Erst danach medizinische Versorgung durchführen<br>• Feuerwehr alarmieren (falls noch nicht initial geschehen)
> 200 ppm	Rückzugsschwelle	**Achtung: CO in gefährlicher Konzentration vorhanden!**<br>• Feuerwehr alarmieren (falls noch nicht initial geschehen)<br>• Betroffenen Bereich räumen und für ein Absenken der CO-Konzentration sorgen (z.B. großflächiges Querlüften) → sofortige Rettung unter Beachtung des Eigenschutzes.<br>• Weitere Maßnahmen danach unter umluftunabhängigem Atemschutz bzw. geeignetem CO-Filtergerät durchführen.<br>• Messwertanzeige des CO-Warngeräts laufend beobachten und Lage ständig neu bewerten.
> 500 ppm	!	**Achtung: Akute Gefährdung durch CO!**<br>Alle Maßnahmen nur unter umluftunabhängigem Atemschutz durchführen.

Tabelle 1: Exposition und Verhaltensweisen, nach [1], [2], [3] und [6]modifiziert

Attachment 1 to: Jüttner B, Busch HJ, Callies A, Dormann H, Janisch T, Kaiser G, Körner-Göbel H, Kluba K, Kluge S, Leidel BA, Müller O, Naser J, Pohl C, Reiter K, Schneider D, Staps E, Welslau W, Wißuwa H, Wöbker G, Muche-Borowski C. S2k guideline diagnosis and treatment of carbon monoxide poisoning. GMS Ger Med Sci. 2021;19:Doc13. DOI: 10.3205/000300, URN: urn:nbn:de:0183-0003009

Literatur:

[1] G. Kaiser, D. Müller, Einsatz von Kohlenstoffmonoxidwarngeräten im Rettungsdienst, Notfall+Rettungsmedizin 2014, 17:141–146

[2] R. Merget; Th. Brüning: Stellungnahme des IPA zum Umgang mit Expositionen gegenüber Kohlenmonoxid, 06.10.2014, persönliche Korrespondenz

[3] Feuerwehr Wiesbaden: Gefährdung durch Kohlenmonoxid im Einsatz. Empfehlungen zur Ausstattung und Einsatztaktik, 2012

[4] CO-Konferenz der Feuerwehr Hamburg, Januar 2015, persönliche Korrespondenzen, Teilnehmerunterlagen

[5] Leitfaden zur Erstellung einer Gefährdungsbeurteilung im Feuerwehrdienst (DGUV Information 205-021), www.publikationen.dguv.de

[6] Hessisches Ministerium des Innern und für Sport, Hessisches Ministerium für Soziales und Integration: Leitfaden CO – Einsatz – Schutz von Einsatzkräften (Feuerwehr und Rettungsdienst) bei Einsätzen mit erhöhtem Kohlenstoffmonoxidgehalt in der Atmosphäre, Stand 20. November 2014

[7] GESTIS-Stoffdatenbank – Gefahrstoffinformationssystem der Deutschen Gesetzlichen Unfallversicherung,www.dguv.de/ifa/stoffdatenbank

Herausgeber

Deutsche Gesetzliche
Unfallversicherung e.V. (DGUV)

Glinkastraße 40
10117 Berlin
Telefon: 030 13001-0 (Zentrale)
Fax: 030 13001-9876
E-Mail: info@dguv.de
Internet: www.dguv.de

Sachgebiet Feuerwehren und Hilfeleistungsorganisationen
im Fachbereich Feuerwehren Hilfeleistungen Brandschutz
der DGUV
