# Supplementary material for: S2k guideline diagnosis and treatment of carbon monoxide poisoning
Source: Ger Med Sci. 2021 Nov 4;19:Doc13. doi: 10.3205/000300 (PMC8607608; doi:10.3205/000300)
Supplement: Declaration of interests and management of conflicts of interest [file GMS-19-13-s-002.pdf]

Declaration of interests and management of conflicts of interest
S2k guideline diagnosis and treatment of carbon monoxide poisoning

The following is a tabular summary of the declarations of interest, as well as the results of the conflict of interest assessment and actions that were decided upon by the guideline group after discussion of the issues and implemented at the consensus conference.

	Consultant or expert activity	Cooperation on a scientific advisory board	Paid lecturing or training activities	Paid authorship or coauthorship	Research projects/conducting clinical studies	Ownership interests (patent, copyright, share ownership)	Indirect interests	Guideline topics affected by COI[1], classification in terms of relevance, impact
Björn Jüttner	No	No	No	No	National Hyperbaric Oxygen Therapy Registry	No	DIVI mandate holder, guideline coordinator<br>Employer: Hannover Medical School<br>Member: DGAI, GRC, EUBS, GTÜM	Diving and hyperbaric medicine<br>Predominant clinical activity in anesthesia, intensive care, and emergency medicine<br>No restriction on function in the guideline group
Thorsten Janisch	No	No	Instructor at HBO Center Euregio Aachen for the training of diving/pressure chamber physicians	Thieme Verlag, book contribution: hyperbaric medicine	No	No	Mandate holder of the DGAI<br>Employer: B.A.D. Gesundheitsvorsorge und Sicherheitstechnik GmbH, physician in advanced training for occupational medicine<br>Member: GTÜM, EUBS, DIVI<br>Freelance physician, HBO Center Euregio Aachen	Diving and hyperbaric medicine<br>Predominant clinical activity in intensive care medicine<br>Moderate restriction on role in level 2 guideline group (abstention on HBOT recommendations)
Johannes Naser	No	No	Instructor at Klinikum Ludwigsburg for the training of diving/pressure chamber physicians	No	No	No	Acting mandate holder of the DGAI<br>Employer: RKH Klinikum Ludwigsburg<br>Member: GTÜM<br>Organization of diving medical course, Klinikum Ludwigsburg<br>Freelance pressure chamber physician, Klinikum Ludwigsburg	Diving and hyperbaric medicine<br>Predominant clinical activity in anesthesia and intensive care medicine<br>Moderate restriction on role in level 2 guideline group (abstention on HBOT recommendations)

Attachment 2 to: Jüttner B, Busch HJ, Callies A, Dormann H, Janisch T, Kaiser G, Körner-Göbel H, Kluba K, Kluge S, Leidel BA, Müller O, Naser J, Pohl C, Reiter K, Schneider D, Staps E, Welslau W, Wißuwa H, Wöbker G, Muche-Borowski C. S2k guideline diagnosis and treatment of carbon monoxide poisoning. GMS Ger Med Sci. 2021;19:Doc13. DOI: 10.3205/000300, URN: urn:nbn:de:0183-0003009

	Consultant or expert activity	Cooperation on a scientific advisory board	Paid lecturing or training activities	Paid authorship or coauthorship	Research projects/conducting clinical studies	Ownership interests (patent, copyright, share ownership)	Indirect interests	Guideline topics affected by COI[1], classification in terms of relevance, impact
Karsten Kluba	No	No	Speaker for state medical association, diagnosis and treatment of carbon monoxide poisoning	No	No	No	Acting Member of the DGAI<br>Employer: University Hospital Leipzig<br>Medical Director Hyperbaric Medical Center UKL<br>Member: GTÜM	Diving and hyperbaric medicine<br>Predominant clinical activity in intensive care medicine<br>Moderate restriction on role in level 2 guideline group (abstention on HBOT recommendations)
Hans-Jörg Busch	No	No	No	No	No	No	Mandate holder of the DGIIN<br>Employer: University Emergency Center Freiburg<br>Member: GRC, DIVI	No restriction on function in the guideline group
Karl Reiter	No	No	No	No	No	No	Mandate holder of the GNPI<br>Employer: Dr. von Haunersches Kinderspital, LMU Hospital Munich<br>Member: ESPNIC	No restriction on function in the guideline group
Gabriele Wöbker	No	No	No	No	No	No	Mandate holder of the DGNI<br>Employer: HELIOS University Hospital Wuppertal<br>Member: BDA, DIVI, DGN, DGNC	No restriction on function in the guideline group
Dietmar Schneider	No	No	No	No	No	No	Acting mandate holder of the DGNI, Emeritus<br>Member: DIVI	No restriction on function in the guideline group
Bernd A. Leidel	No	No	No	No	No	No	Mandate holder of the DGINA<br>Employer: Charité - Universitätsmedizin Berlin	No restriction on function in the guideline group
Harald Dormann	No	No	No	No	No	No	Acting mandate holder of the DGINA<br>Employer: Klinikum Fürth	No restriction on function in the guideline group

	Consultant or expert activity	Cooperation on a scientific advisory board	Paid lecturing or training activities	Paid authorship or coauthorship	Research projects/conducting clinical studies	Ownership interests (patent, copyright, share ownership)	Indirect interests	Guideline topics affected by COI[1], classification in terms of relevance, impact
Guido Kaiser	No	No	Recognition and treatment of poisoning	Recognition and treatment of poisoning	BMBF and the German Fire Protection Association: personnel and material resources, fatalities, and serious injuries in fires	No	Mandate holder of the GIZ-North<br>Employer: University Medical Center Göttingen	Activity in research, emergency medicine, poisoning<br>Minor restriction on function in the guideline group for level 1 (working group leadership)
Andreas Callies	No	No	No	No	No	No	Mandate holder of the BAND<br>Employer: Klinikum Links der Weser	No restriction on function in the guideline group
Oliver Müller	No	No	Lecturer at the University of Leipzig, Traunstein pressure chamber, Ludwigsburg clinic for the training of diving /pressure chamber physicians	No	No	No	Mandate holder of the DGAI<br>Employer: Vivantes Klinikum im Friedrichshain<br>Medical Director, Hyperbaric Oxygen Therapy and Diving Medicine Division<br>Member: GTÜM, DIVI, EUBS	Diving and hyperbaric medicine<br>Predominant clinical activity in emergency and intensive care medicine<br>Minor restriction on function in the guideline group for level 1 (working group leadership)
Wilhelm Welslau	No	No	Lecturer at Haux-Life-Support for the training of pressure chamber personnel, as well as at BG-Klinik Murnau and taucherarzt.at for the training of diving/pressure chamber physicians	No	No	No	Deputy mandate holder of the GTÜM<br>Employer: self-employed occupational physician<br>Member: GTÜM, ÖGTH, ECHM	Diving and hyperbaric medicine<br>Moderate restriction on in level 2 guideline group (abstention on HBOT recommendations)
Hella Körner-Göbel	No	No	No	No	No	No	Mandate holder of the ÄLRD<br>Employer: HELIOS University Hospital Wuppertal<br>Member: BAND, AGNNW	No restriction on function in the guideline group

Holger Wißuwa	No	No	Case presentation: carbon monoxide accident	No	No	No	Acting mandate holder of the ÄLRD<br>Employer: Evangelisches Krankenhaus Herne<br>Member: BAND, AGNNW	Preclinical emergency medicine<br>Minor restriction on function in the guideline group for level 1 (working group leadership)
Carsten Pohl	No	No	Indications and contraindications of HBOT	No	No	No	Additional author of this guideline (no voting rights)<br>Employer: AMEOS Klinikum Bernburg<br>Member of the Guideline Group until 12/2017	Hyperbaric medicine<br>Predominant clinical activity in emergency and intensive care medicine<br>Minor restriction on function in the guideline group for level 1 (working group leadership)
Enrico Staps	No	No	No	No	No	No	Additional author of this guideline (no voting rights)<br>Employer: Bundeswehrkrankenhaus Ulm<br>Physician at a hospital-operated pressure chamber<br>Member: BDA, AGBN	Hyperbaric medicine<br>Predominant clinical activity in emergency and intensive care medicine<br>Minor restriction on function in the guideline group for level 1 (working group leadership)
Stefan Kluge	No	No	No	No	No	No	Additional author of this guideline (no voting rights)<br>Employer: University Medical Center Hamburg-Eppendorf<br>Member: DGP, DIVI	Intensive care medicine<br>Minor restriction on function in the guideline group for level 1 (working group leadership)
Cathleen Muche-Borowski	No	No	No	No	No	No	Moderation of the guideline group (no voting rights)<br>Employer: University Hospital Hamburg-Eppendorf	No restriction on function in the guideline group

[1]Only those statements for which a thematic reference to the guideline was determined in the guideline group after discussion and evaluation of the facts fully disclosed according to the AWMF form were transferred to the tabular summary here. The complete statements are held in the guideline secretariat
[2] Alternatively, simply "Yes" can be entered and the naming of the companies can be waived
